# Risk stratification of pulmonary toxicities in the combination of whole lung irradiation and high-dose chemotherapy for Ewing sarcoma patients with lung metastases: a review

**DOI:** 10.1007/s00066-020-01599-8

**Published:** 2020-03-12

**Authors:** Sergiu Scobioala, Hans Theodor Eich

**Affiliations:** Department of Radiotherapy and Radiooncology, Universitätsklinikum Münster, Albert-Schweitzer-Campus 1, Gebäude A1, 48149 Muenster, Germany

**Keywords:** Ewing sarcoma, Lung metastases, Chemotherapy, Radiotherapy, Lung side effects

## Abstract

**Background:**

Whole lung irradiation (WLI) represents an important part of multimodal therapy in Ewing sarcoma (EwS) patients diagnosed with pulmonary metastases. This review discusses pulmonary toxicity in EwS patients with pulmonary metastases treated with WLI, who received different modes of high-dose chemotheray (HD-Cth).

**Methods:**

Literature was compiled using the Cochrane Library, PubMed database, and the National Institute of Health (NIH) clinical trials register. Relevant patient information, including nature of HD-Cth, acute and late lung toxicities, and pulmonary function disorders, was selected from the above databases.

**Results:**

Nine reports with a total of 227 patients, including 57 patients from a single randomized trial were included in this review. No acute or chronic symptomatic pulmonary toxicities were observed in patients that received WLI after HD busulfan-melphalan (HD-Bu/Mel), but 8% of these patients were diagnosed with asymptomatic restrictive lung disease. Grade 1 or 2 acute or chronic lung adverse effects were observed in up to 30% of patients that received WLI after HD treosulfan/Mel (HD-Treo/Mel) or HD etoposide (E)/Mel. Interstitial pneumonitis was present in 9% of patients treated concurrently with E/Mel and total body irradiation (TBI) with 8 Gy. Radiation doses as well as time between HD-Cth and WLI were both identified as significant risk factors for pulmonary function disorders.

**Conclusion:**

The risk of adverse lung effects after WLI depends on several factors, including cumulative radiation dose and dose per fraction, HD-Cth regimen, and time interval between HD-Cth and WLI. A cumulative radiation dose of up to 15 Gy and a time interval of at least 60 days can potentially lead to a reduced risk of pulmonary toxicities. No evident adverse lung effects were registered in patients that received simultaneous therapy with HD-Cth and TBI. However, pulmonary function testing and lung toxicity reports were lacking for most of these patients.

## Introduction

Despite a lack of prospective trials, present data show whole lung irradiation (WLI) to be an important part of curative therapy for pediatric and young adult patients with pulmonary manifestations of Ewing sarcoma (EwS) [1–5]. Most importantly, the use of WLI in these patients resulted in a marginal trend towards better survival [1–5]. According to the multicenter cooperative EwS studies (CESS), WLI should complement multimodal therapy in pediatric EwS patients with complete radiological remission of pulmonary metastases following polychemotherapy or after the resection of residual pulmonary metastases [6].

However, these studies focussed on survival outcome, not on treatment-related toxicities. This has changed with a newly established European Ewing Tumor Working Initiative of National Groups Ewing Tumor Studies 1999 (EURO‑E.W.I.N.G. 99) and records of acute and late radiation-related side effects have been collected. Published data indicate an acceptable tolerability of WLI in children, adolescents, and young adults, also in combination with conventional chemotherapy (Cth) [1–3, 7, 8].

However, tolerance of WLI combined with high-dose Cth (HD-Cth) has not been sufficiently analyzed in published multicentre studies researching therapy and survival in EwS patients with pulmonary metastases [1, 2, 5, 9]. This systematic review evaluates the risk stratification of pulmonary function disorders, as well as radiogenic acute and late lung toxicities in the combined use of HD-Cth and WLI in primary and relapsed EwS patients exhibiting lung metastases.

## Material and methods

### Study selection

**Fig. 1:**
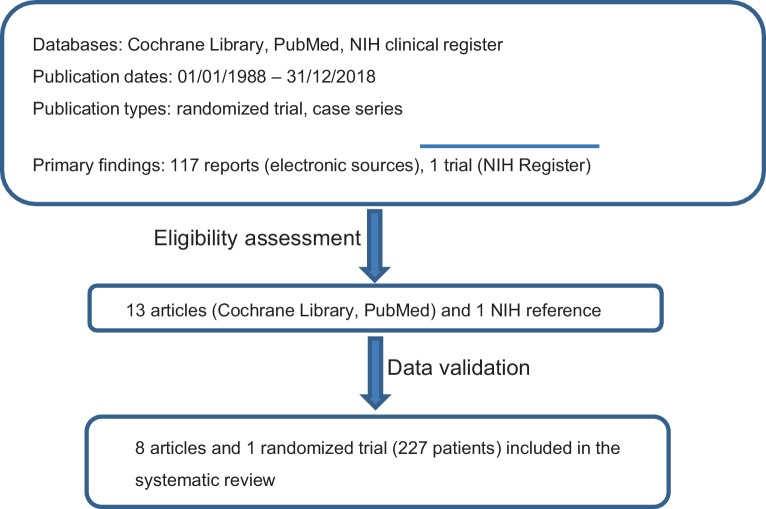
Flow chart of report selection process. The database resources included: Cochrane Library, PubMed, and the National Institutes of Health clinical trials register (https://clinicaltrials.gov/). The following search terms were used: Ewing Sarcoma, pulmonary metastases, whole lung irradiation, high-dose chemotherapy, pulmonary side effects

This systematic review was structured according to the preferred reporting items for systematic reviews and meta-analyses (PRISMA) reporting guidelines [10]. The seven-step model was used to perform the literature search, as described in detail by Onwuegbuzie & Friels and Williams [11, 12]. The respective flow chart is presented in Fig. 1. Studies on WLI combined with Cth or HD-Cth that recruited patients with primary EwS of any localization exhibiting pulmonary metastases or patients with isolated pulmonary relapse were analysed. Concurrent or sequential Cth regimens given in mono- or polychemotherapy settings were included. Published trials were identified using the Cochrane Library and PubMed database. In addition, the National Institutes of Health (NIH) clinical trials register (https://clinicaltrials.gov/) was searched to select appropriate clinical trials. Case reports, reviews and planning studies were excluded. The following phrases were used for eligibility criteria: “articles must be peer reviewed”; “articles must be less than thirty years old”; and “article must contain qualitative and/or quantitative analyses.” Only articles published in English were considered. When searching for articles, the authors directly specified all terms used, i.e., Ewing Sarcoma, pulmonary metastases, whole lung irradiation, HD-Cth, and pulmonary side effects.

### Data extraction and study quality assessment

Literature search, selection of studies, and data extraction were separately performed by two trained and certified radiation oncologists (SS). The senior author (HTE) validated the results and resolved any discrepancies concerning data assessment.

The following patient information was independently extracted from the databases: age, sex, radiation technique, fraction and cumulative radiation dose for WLI, additional boost in case of chest wall primary EwS, resection of pulmonary lesions, mode of HD-Cth, post-treatment follow-up, acute and late lung toxicities, and pulmonary functional test.

Quality assessment of the selected studies was performed using the Cochrane risk-of-bias tool [13].

### Statistical analysis

Due to significant heterogeneity within a variety of factors across the studies, including the age of patients, radiotherapy (RT) regimen, mode of HD-Cth, methods of side-effect estimation, and patient follow-up, a Mantel-Haenszel random-effect model was used to estimate the mean distribution of acute and late lung toxicity across the studies. The Cox proportional hazard regression analysis and the Pearson correlation test were used to determine the risk of lung and non-lung-related adverse effects analyzing the following variables: age, sex, cumulative radiation dose, HD-Cth regimen, time interval between HD-Cth and WLI, RT technique, and radiation boost (Table 2). Statistical analysis was carried out with the SPSS program (SPSS for Windows, Version 24.0; IBM Corp., Armonk, NY, USA). A *p*-value of <0.05 was set.

## Results

### Literature search

A total of 117 reports were initially screened and 13 were then assessed for eligibility. Nine studies with a total number of 227 patients were included in the review (Fig. 1, Table 1). One study was a randomized controlled single-arm trial [14], while all other studies were retrospective analyses [1, 5, 15–20]. Most of the retrospective series were considered to have a low risk of selection bias. The earliest article was published by Miser et al. in 1988 and the latest by Scobioala et al. in 2018 [5, 18]. The studies were reported both by radiation oncology and pediatrics departments (Table 1).

**Table 1:** Therapy-related toxicity outcome

Author/year	HD-Ctx+ WLI (pts)	Cumulative/ fraction dose (Gy)	Boost^d^ (pts/Gy)	Resection of lung lesions (pts)	Primary Ctx	HD-Ctx (mode/pts)	Interval between HD-Ctx and WLI (days)	Lung function disorder^a^ (severity/pts)	Acute lung toxicity^b^ (pts/grade)	Late lung toxicity^b^ (pts/grade)	Other radiation-related toxicities^c^	Death related to therapy (pts)
Luksch/2012 [14]	57	15/1.5 > 14y. 12/1.2 < 14y.	n.a.	6	VAI_CE/VAC_IE	Bu/Mel (57)	60–90	Mild: 1 Moderate: 2 Severe: 1	0	0	0	1
Paulussen/ 1998 [1]	3	18/1.5 > 14y. 15/1.5 < 14y.	n.a.	n.a.	VACA or VAIA or EVAIA	Bu/Mel (3)	n.a.	Severity n.a./7	1/1	1/1	1	2
Scobioala/2018 [5]	32	18/1.5 > 14y. 15/1.5 < 14y.	9 (45)	5	EVAIA or VIDE	Treo/Mel (27) E/Mel (5)	30–60	Mild: 5 Moderate: 3 Severe: 0	5/1 2/2	4/1 1/2	0	0
Burdach/2003 [15]	26	8/2 × 1.5/d.^e^	n.a.	0	EVAIA or VIDE	E/Mel (26)	Concurrently	n.a.	2/2	0	2	6
Pape/1999 [16]	39	10/2 × 1.5/d.^e^, or 18/1.5 > 15y. 15/1.5 < 15y.	n.a.	n.a.	EVAIA	E/Mel (39)	Concurrently (17 pts) Sequentially (22 pts)	n.a.	n.a.	n.a.	n.a.	6
Czyzewski/1999 [17]	9	12/2 × 2/d.^e^ (6 pat.) 15/1.5 (1 pt.) 12.8/2 × 1.6/d. (1 pt.)	n.a.	3	n.a.	E/Mel (9)	Concurrently (6 pts) Sequentially (3 pts)	n.a.	0	0	n.a.	1
Miser/1988 [18]	21	8/2 × 2/d.^e^	2 (55–60)	0	n.a.	VADRIAC (21)	Concurrently	n.a.	0	0	n.a.	0
Burdach/1993 [19]	17	8/2 × 1.5/d.^e^	1 (30) 1 (50)	4	VAIA or EVAIA	E/Mel (17)	Concurrently	–	2/2	1/3	0	1
Meyers/2001 [20]	23	8/2 × 2/d.^e^	–	–	VACA	E/Mel (23)	Concurrently	n.a.	0	0	1	5

*pt(s)* Patient(s); *WLI* whole lung irradiation; *Cth/HD-Ctx* chemotherapy/high-dose chemotherapy; *Gy* gray; *n.a.* detailed information is not available from the report; *Treo* treosulphan; *Mel* melphalan; *E* etoposide; *C* cyclophosphamide; *V* vincristine; *I.* ifosfamide; *A* adriamycin; *D* doxorubicin

^a^ Pulmonary function disorders were examined by pulmonary function testing

^b^ Acute and late lung toxicities graded according to the Radiation Therapy Oncology Group and European Organization for Research and Treatment of Cancer toxicity criteria (15)

^c^ Not pulmonary adverse effects: dysphagia, esophagitis, cardiac disorders, etc..

^d^ Additional boost for primary chest wall Ewing sarcoma

^e^ Consolidating therapy that includes total body irradiation and high-dose chemotherapy for primary metastatic or relapsed Ewing sarcoma patients

### Treatment modalities

In two studies, HD-Cth was given as consolidation treatment after maintenance Cth and before WLI [5, 14]. Lucksch et al. used HD-Cth with busulfan/melphalan (Bu/Mel) in 57 patients with primary pulmonary or single bone metastases with an interval of 60–90 days before WLI [14]. The cumulative RT ranged from 12 to 15 Gy, while the daily dose per fraction was 1.2–1.5 Gy (Table 1). Scobioala et al. reported on HD-Cth with either treosulfan/melphalan (Treo/Mel) (27 patients) or etoposide/melphalan (E/Mel) (five patients) in EwS patients with isolated pulmonary relapse. The WLI was applied in 30–60 days after HD-Cth [5]. A total RT dose ranged between 15 and 18 Gy with a daily fractionation of 1.5 Gy (Table 1).

In two series performed by Pape et al. and Czyzewski et al., total body irradiation (TBI) was administrated concurrently or sequentially to HD-E/Mel in primary metastatic or relapsed EwS patients [16, 17]. A total RT prescribed dose on the lungs ranged from 10 to 12 Gy for simultaneous use, or from 15 to 18 Gy for a sequential regimen. In one patient, a total dose of 12.8 Gy was delivered with a single dose of 1.6 Gy twice daily (Table 1).

In three other studies, a cumulative dose of 8 Gy to the lungs was delivered within TBI for primary metastatic or relapsed EwS patients simultaneously to E/Mel or VADRIAC HD-Cth by using of 1.5 Gy or 2 Gy delivered twice daily (Table 1; [18–20]).

In the setting of pulmonary and/or pleural manifestations of EwS relapse, Scobioala et al. analyzed the effect of a sequential boost on residual tumor localized in the chest wall or thoracic vertebra [5]. There were no data concerning thoracic boost toxicity in other studies.

In four studies on a total of 14 patients, resection of lung lesions was performed before HD-Cth and WLI [5, 14, 17, 19]. In two reports, data on metastasectomy of lung nodules were not available (Table 1; [1, 16]).

### Analysis of adverse effects

The toxicity results are shown in detail in Table 1.

Follow-up pulmonary function test (PFT) data were available in three studies for 65 of the 227 patients that received WLI. Lung function impairments were described in detail by Scobioala and colleagues [5]. However, in that particular study, PFT acquisition time relative to treatment time was not reported. In other reports, PFTs were only performed in small numbers of patients [1, 14]. Generally, restrictive lung disease was found in 20 patients (31%) that had been treated sequentially with HD-Cth and WLI. Of those, severe lung function disorders were observed in two (8%) of 24 patients that received WLI after HD-Bul/Mel. The patients treated with Treo/Mel or E/Mel and consolidating WLI did not develop severe pulmonary functional disorders.

Pre-treatment PFTs were reported only in a paper by Scobioala and colleagues [5]: In five patients (8%), mild or moderate lung impairment was diagnosed without further worsening after WLI. No significant differences in pulmonary function were found during a median follow-up of 3 years in any of the 27 patients. Acute or chronic pulmonary toxicity grades 1 and 2 were observed in seven of 27 patients (26%) with pulmonary relapsed EwS that received WLI after HD-Cth with Treo/Mel or E/Mel (Table 1). Interstitial pneumonitis was diagnosed in four of 43 patients (9%) treated concurrently with E/Mel and TBI with 8 Gy [15, 19]. Across the studies, non-lung-related adverse effects were mentioned in only four patients (1.8%) that developed dysphagia or cardiac arrhythmia. No growth delay or skeletal abnormalities were reported in any studies. Therapy-related death was registered in 23 of 227 patients (10%). However, in most cases, the mortality was associated with the HD-Cth regimen rather than with the WLI: The most common causes of death were hemorrhagic or infectious complications, and four patients died from respiratory complications [1, 19]. Second malignancies noted included acute myeloid leukemia (three patients), myelodysplastic syndrome (three patients), and liposarcoma (one patient) [15].

Risk factors that might influence therapy-related toxicity in HD-Ctx and WLI regimens were also analyzed (Table 2). Regarding the impact of radiation dose on pulmonary function disorders, the 5% level of significance was reached for 8–12 Gy and 15 Gy in the Pearson correlation test (*p* = 0.04 and *p* = 0.03, respectively), but failed in the Cox proportional hazard regression analysis. The data for 18 Gy was not eligible for statistical evaluation. The time interval of 30–60 days and 60–90 days between HD-Cth and WLI demonstrated a significant impact on pulmonary function disorders in all tests. Significantly higher rates of therapy-related death were found in patients treated simultaneously with E/Mel and TBI with 8 or 12 Gy. Gender, age, and radiation technique showed no significant impact on therapy-related pulmonary adverse effects or death of patients. No relation between an increase in pulmonary toxicities and resection of pulmonary tumor rest was observed in the study by Scobioala et al. [5]. In other studies, findings on lung nodule surgery were rarely reported. The data on radiation boost for thoracic EwS were not eligible for the assessment (Tables 1 and 2).

**Table 2:** Prognostic factors for therapy-related toxicities in patients treated with high-dose chemotherapy and whole lung irradiation

Parameter	Lung function disorder		Acute lung toxicity		Late lung toxicity		Other toxicities		Death related to therapy	
	%/N*	P**	%/N*	P**	%/N*	P**	%/N*	P**	%/N*	P**
*Sex*										
Female	11.3/45	–	8.3/37	–	6.4/37	–	3.6/51	–	11.2/87	–
Male	14.2/52	0.27	12.6/43	0.21	9.2/43	0.32	4.7/59	0.45	15.8/119	0.37
*Median age*										
<15	8.9/124	–	9.7/124	–	5.6/124	–	3.2/124	–	16.7/124	–
≥15	6.8/82	0.32	7.3/82	0.22	4.1/82	0.14	4.4/82	0.27	18.1/82	0.13
*Total radiation dose*										
8–12 Gy	8 (35)	–	4.1 (97)	–	1.3/74	–	3.2/93	–	17/101	–
15 Gy	9.8/61	0.09	8.1/61	0.18	5.5/61	0.17	4.1/61	0.42	3.2/61	0.51
18 Gy	16 (25)	–	12/25	–	8/25	–	4/25	–	4/25	–
*HD-Cth*										
Bu/Mel	6.7/60	–	1.7/60	–	1.7/60	–	1.7/60	–	5/60	–
Treo/Mel	3.8 /27	0.24	4.1/27	0.25	2.2/27	0.09	0/27	0.32	0/27	0.22
E/Mel	n.a.	–	8.6/58	0.17	3.4/58	0.34	5.2 /58	0.27	19.1/99	0.001
*Interval between HD-Ctx and WLI (days)*										
0–30 ^b^	n.a.	–	6.1 (65)	–	1.5(65)	–	4.6/72	–	18.2/104	–
30–60	9/32	0.005	7.8/32	0.17	5.6/32	0.12	0/32	0.10	0/32	0.001
60–90	11/57	0.009	5.3/57	0.21	3.6/57	0.27	0/57	0.13	1.7/57	0.002
*RT technique*										
AP/PA	11.9/92	–	12.2/98	–	7.1/98	–	4.5/89	–	16/137	–
IMRT ^c^	14.3/7	–	14.3/7	–	0/7	–	0/7	–	0/7	–
*Radiation boost*^a^										
Yes	8.3/13	–	16.6/13	–	0/13	–	0/13	–	8.3/13	–
No	n.a.	–	n.a.	–	n.a.	–	n.a.	–	n.a.	–

*WLI* whole lung irradiation; *Cth/HD-Ctx* chemotherapy/high-dose chemotherapy; *Bu/Mel* busulfan/melphalan; *Treo* treosulfan; *Mel* melphalan; *E* etoposide; *AP/PA* anterior-posterior/posterior-anterior; *IMRT* intensity-modulated radiation therapy; *RT* radiotherapy; *n.a* detailed information is not available from the report

N* number of patients with available data; P** 5% level of significance obtained by Cox proportional hazard regression analysis

^a^ Additional boost for primary chest wall EwS

^b^ The time interval of 0–30 days represents a consolidating therapy that includes total body irradiation and high-dose chemotherapy for primary metastatic or relapsed EwS patients

^c^ Very limited number of patients with IMRT (*n* = 7), statistical comparison was not meaningful

## Discussion

A systemic analysis is presented here of WLI-related pulmonary toxicities with radiation applied sequentially or concurrently to HD-Cth in EwS patients with primary lung metastases or pulmonary relapse.

Past multicenter studies including CESS-81, CESS-86, and European Intergroup (EI)CESS-92 demonstrated a therapeutic benefit for WLI in pediatric EwS patients with primary lung metastases [1, 2, 6, 7]. Improved local control of pulmonary disease as well as a marginal trend toward better progression-free survival make WLI an important treatment option that should complement multimodal therapy in patients with lung relapse of EwS [5]. Published data indicate acceptable pulmonary toxicity for WLI in children, adolescents, and young adults in combination with maintenance Cth [1–3, 7, 8].

The randomized Euro‑E.W.I.N.G 99 study compared HD-Cth with Bu/Mel versus conventional Cth combined with WLI in EwS patients with pulmonary metastases.

The combination of HD-Cth with WLI in EwS patients with primary pulmonary metastases or pulmonary relapse was performed in several studies [1–3, 5, 6]. The cumulative doses on the lungs varied from 14 to 18 Gy, similarly to the combination with conventional Cth.

Luksch and colleagues combined HD-Bu/Mel and WLI in a large number of EwS patients (*n* = 57) with lung-only metastases and no lung function impairment after HD-Cth [14]. The radiation dose was age-dependent and varied from 12 to 15 Gy, which was applied 60 or 90 days after HD-Cth. Higher risk of additional pulmonary toxicities has limited the use of HD-Bu/Mel and WLI in other cooperative studies [21–23]. This is the first prospective single-arm study that reports acceptable pulmonary toxicity for the combination of Bu/Mel and WLI. However, the cumulative radiation dose with a maximum of 15 Gy was lower than in other trials, where WLI with a maximum dose of 18 Gy was combined with HD-Treo/Mel or HD-E/Mel (Table 1). Furthermore, the interval between HD-Bu/Mel and WLI was longer compared to other therapy regimens (60–90 days versus 30–60 days, respectively). And finally, pulmonary function was carefully analyzed to pre-select patients eligible for therapy with HD-Bu/Mel and additional WLI. Based on this study, a cumulative radiation dose of 15 Gy and a time interval of at least 60 days might be recommended for WLI after HD-Bu/Mel in high-risk EwS patients with no lung function injury.

Combination therapy of HD-Treo/Mel or HD-E/Mel and WLI is widely used in patients with primary metastatic or relapsed EwS [5, 15–17, 19, 20]. Generally, the reports presented in Table 1 demonstrated a low rate of lung function disorders or lung toxicities using a radiation dose of 18 Gy applied 8–10 weeks after HD-Treo/Mel or HD-E/Mel. However, the lack of regular post-treatment PFTs and documentation of adverse lung effects, which are most problematic in the studies analyzing simultaneous HD-Cth and WLI, could lead to an underestimation of lung toxicities (Table 1).

In most analyzed studies, survival and not therapy-related adverse effects were the main outcome parameters to be evaluated. Thus, findings on tolerability of WLI after HD-Cth remain extremely limited [5, 7, 9, 14].

No severe pulmonary function disorders or acute lung toxicities were reported after WLI when applied sequentially following HD-Treo/Mel or HD-E/Mel in analyzed studies (Table 1). Paulussen et al. described one of three patients that died due to fulminant acute pneumonitis after therapy with HD-Bu/Mel and WLI [1]. No correlation between pulmonary side effects and specific HD-Cth modality was apparent across the studies.

Concomitant use of HD-Cth and TBI was reported in a number of publications [15–20]. The cumulative radiation dose on the lungs varied between 8 and 12 Gy with fractions between 1.5 or 2 Gy applied once or twice daily (Table 1). HD-E/Mel was used in most of these studies. As reported by Horowitz et al. and Ladenstein et al., myeloablative therapy with concomitant use of HD-Cth and TBI did not improve survival outcomes in metastatic EwS and rhabdomyosarcoma patients [24, 25]. In contrast, Gryzewski et al. and Burdach et al. reported improved survival for HD-Cth and 12 Gy TBI compared to HD-Cth alone [17, 19].

Regardless, a simultaneous combination of HD-Cth and TBI may contribute to high toxicity and increased rates of therapy-related death, as demonstrated in Table 1. Due to insufficient data on post-treatment PFTs in these patients, the authors can only suspect a higher risk of lung function disorders following this therapy. They also assume that the low reported rate of registered acute and late pulmonary toxicities is most likely related to the small number of patients that underwent post-treatment lung function analysis. Thus, comparable survival results but possibly reduced rates of lung toxicities and therapy-related death for consolidating WLI compared to concomitant TBI cast doubt on the role of TBI in the treatment of patients with metastatic EwS.

The treatment regimen combining HD-Cth and WLI and not a cumulative radiation dose on the lungs is more an important prognostic factor for the development of pulmonary toxicities. This suggestion is supported by significantly higher rates of death after simultaneous use of HD-E/Mel and TBI with 8 Gy or 12 Gy compared to patients treated sequentially with HD-E/Mel and WLI delivering doses of 15 Gy or 18 Gy (Tables 1 and 2). A radiation dose of 20 Gy in 2‑Gy fractions can cause severe lung toxicities in 12.2% of patients, as demonstrated by Trifaud et al. [26]. Use of the same radiation dose and the same fractionation by Burger et al. was well tolerated without severe toxicities in 140 irradiated patients [27]. The impact of radiation dose in relation to fractionation on the development of pulmonary side effects was not evident in the present analysis.

The impact of pulmonary tumor rest resection following HD-Cth on radiogenically induced lung toxicities was analyzed in detail in the study by Scobioala et al. in patients with pulmonary relapsed EwS. The study showed no increased risk for lung adverse effects after surgery of the tumor rest [5]. In contrast, some authors observed a correlation between thoracic surgery and the development of pulmonary side effects following induction Cth (VAIA or VIDE) and WLI [2, 28].

In other studies, data concerning lung nodule surgery was available in only a few patients and, for this reason, not eligible for prognostic assessment (Tables 1 and 2). Similarly, the impact of a radiation boost to the chest wall on adverse lung effects remains unclear due to a limited number of patients treated with a boost in this setting (Tables 1 and 2). However, an additional boost to the thorax region should be considered a risk factor for radiogenically induced pneumonitis and pulmonary fibrosis. Gender, age, and radiation technique revealed no prognostic significance concerning therapy-related pulmonary or non-pulmonary adverse effects (Table 2). The authors found no difference in the frequency and severity of lung toxicities after HD-Cth and WLI between patients under and over 15 years of age (Table 2). These findings are supported by those of Casey et al., who observed acceptable tolerance of WLI in adults with pulmonary metastatic EwS [3].

Late toxicities such as underdevelopment of the chest wall in children or radiation-induced secondary malignancies were not analyzed in this setting. Generally, the risk of chest wall underdevelopment in children resulting in reduced lung volumes is increased after WLI, as described by Benoist et al. in patients with Wilm’s tumour [29].

Evidence of side effects after radiotherapy in children and adolescents is recorded in a specific National Registry in Germany [30, 31].

The limitations of this systematic analysis include: 1) Deviations in the treatment protocols, both for systemic and local treatment between institutes, 2) different selection criteria for the studies, 3) data used to evaluate lung toxicities are incomplete, and 4) relatively short follow-up in most of the analyzed studies. The deviations in the therapy protocols are related to different study designs. Lack of treatment homogeneity in terms of different HD-Cth and WLI regimens, including the nature of HD-CTh, methods of side effect estimation, the cumulative radiation dose on the lungs, dose per fraction, and different interval between HD-Cth and WLI, could account for the underestimation of the risk for lung toxicities.

Given these findings, the risk of pulmonary toxicities after HD-Cth and WLI in EwS patients with primary lung metastases or pulmonary relapse should be estimated in a prospective randomized trial. Additionally, less toxic but more effective radiotherapy and HD-Cth regimens should be identified. For instance, the use of ultra-fractionated WLI with a fraction dose of <0.5 Gy could be evaluated for combination with HD-Cth based on the hyper-radiation sensitivity phenomenon [32–34]. Based on preclinical studies, Dey et al. and Gupta et al. suggested an increased Cth-related apoptotic effect in combination with low-fraction radiotherapy [35, 36]. At the same time, RT with a lower fraction dose may potentially be less toxic. Given the poor prognosis with metastatic or relapsed EwS disease, quality of life should be of emphasized importance in the indication of WLI after HD-Cth. Also, radiotherapy approaches including intensity-modulated radiotherapy (IMRT) and proton therapy should be evaluated for WLI, which may potentially improve the risk of acute and late pulmonary or extrapulmonary adverse effects. Thus, superior sparing of the heart may be achieved by using a novel IMRT prescription dose, termed simultaneous integrated protection for organ at risk, as shown by Mazzola et al. for stereotactic body radiotherapy of central lung malignancies [37]. Bousabarah et al. applied a radiomic analysis of the gross tumor volume in lung cancer patients treated with stereotactic radiotherapy to predict radiation-induced lung fibrosis [38]. Dhami et al. used anatomic and perfused lung dosimetry for the risk stratification of radiation pneumonitis in patients undergoing definitive thoracic radiation [39].

## Conclusion

Few relevant conclusions concerning pulmonary toxicity after combination therapy with HD-Cth and WLI can be drawn due to heterogeneity in study designs and reporting of results. The only reliable evidence relates to the increased incidence of death in patients simultaneously receiving HD-Cth and TBI with a minimum lung radiation dose of 8 Gy. Meanwhile, the risk of lung disorders and lung toxicities is negligible in patients that received HD-Cth and WLI sequentially, including patients treated with HD-Bu/Mel. Based on the analyzed reports, it is difficult to stratify the patients that may be susceptible to pulmonary toxicity, as well as to define the relationship between HD-Cth and WLI regimens and pulmonary complications. For this reason, the risk of lung toxicities should be individually evaluated based on, e.g., the presence of preexisting lung disorders, use of high cumulative radiation doses, or shortened interval between HD-Cth and WLI.
